# Macaques can predict social outcomes from facial expressions

**DOI:** 10.1007/s10071-016-0992-3

**Published:** 2016-05-07

**Authors:** Bridget M. Waller, Jamie Whitehouse, Jérôme Micheletta

**Affiliations:** Department of Psychology, Centre for Comparative and Evolutionary Psychology, University of Portsmouth, Portsmouth, UK

**Keywords:** Facial expression, Communication, Facial signals, Primates, Emotion

## Abstract

There is widespread acceptance that facial expressions are useful in social interactions, but empirical demonstration of their adaptive function has remained elusive. Here, we investigated whether macaques can use the facial expressions of others to predict the future outcomes of social interaction. Crested macaques (*Macacanigra*) were shown an approach between two unknown individuals on a touchscreen and were required to choose between one of two potential social outcomes. The facial expressions of the actors were manipulated in the last frame of the video. One subject reached the experimental stage and accurately predicted different social outcomes depending on which facial expressions the actors displayed. The bared-teeth display (homologue of the human smile) was most strongly associated with predicted friendly outcomes. Contrary to our predictions, screams and threat faces were not associated more with conflict outcomes. Overall, therefore, the presence of any facial expression (compared to neutral) caused the subject to choose friendly outcomes more than negative outcomes. Facial expression in general, therefore, indicated a reduced likelihood of social conflict. The findings dispute traditional theories that view expressions only as indicators of present emotion and instead suggest that expressions form part of complex social interactions where individuals think beyond the present.

**Electronic supplementary material:**

The online version of this article (doi:10.1007/s10071-016-0992-3) contains supplementary material, which is available to authorized users.

## Introduction

When Darwin first described the *expressionsofemotion* of humans and other animals, he proposed that facial expressions may not always be functional communicative signals (Darwin 1872) and thus set the scene for approaching facial expressions more as by-products of feeling states. Classic studies have followed this lead and conceptualized facial expressions largely in terms of the proximate emotional state of the sender only (Ekman et al. 1969). Paradoxically, this approach is at odds with modern evolutionary theory which strives to understand both the ultimate and proximate function of behaviour (Tinbergen 1963). As the basic premise of modern evolutionary science is that ultimate function is necessary for overt behaviours to evolve, this leaves a large gap in the literature. Calls for a more adaptationist approach to facial expressions (Schmidt and Cohn 2001; Fridlund 1994) have since been well received, but empirical demonstration of how individuals benefit from exposing their own and others’ internal states via the face is lacking. Indeed, it could even be argued that revealing emotions and motivations to others have the potential to be maladaptive if they expose vulnerability and weakness (Waller et al. 2014). In order to understand why facial expressions abound in the animal world, therefore, it is vital to establish what advantage is gained from using facial expressions within social interactions.

There is some experimental evidence that observing the facial expressions of others (conspecifics and humans) allows primates to adjust their decisions about things that they themselves cannot see (Morimoto and Fujita 2011; Buttelmann et al. 2009). In these studies, subjects approached or avoided hidden items differentially depending on whether a demonstrator viewed the items with a positive or negative expression. Providing information about unseen parts of the physical environment could indeed be useful, but in most cases, the target of the facial expression is visible to others simultaneously to the facial expression itself. Also, facial expressions tend to be directed not to inanimate physical objects, but instead to conspecifics during social interaction (Liebal et al. 2014).

Facial mobility correlates with group size in primates (Dobson 2009), implying that facial expression must have some overall social function such as solidifying social cohesion in a similar way to vocalizations (McComb and Semple 2005) and grooming (Dunbar 1993). We propose that decoding the facial expression of others could be highly adaptive if this allows the observer to anticipate what action the actor will perform next, and how the actor will interact with others. For example, observers could adjust their own behaviour accordingly if they can anticipate forthcoming conflict in the group. Thus, the facial expressions of others act as pointers to potential actions, as signals of the actors’ ‘action readiness’ (Frijda 2007). Importantly, predicting the likely actions of others *before* they commit to a specific action allows all parties to avoid conflict, thus having an overall social cohesion function and reducing social instability.

The crested macaque (*Macacanigra*) is an ideal species to begin testing the adaptive function of facial expression, as it is equipped with one of the largest and richest repertoires of facial expression in the genus *Macaca* (Dobson 2012). The elaboration of a communicative repertoire in this species has been hypothesized to be a consequence of their increased social tolerance, which increases the need for mechanisms to mitigate uncertainty in social interactions (Dobson 2012). Thus, their increased repertoire is likely to have evolved alongside a sophisticated understanding of others’ facial expressions. Recent experimental data have shown that they can categorize conspecific facial expressions as discrete units from both still pictures and video, even when they appear very similar to human observers (Micheletta et al. 2015).

In the current study, we used a series of experiments with touchscreen-trained crested macaques to explore whether macaques can use the information present in the faces of conspecifics to predict how social interactions will progress. Using a novel experimental design, we tested whether the type of facial expression observed in others causes subjects to predict whether a neutral approach will end in one of two categories of behaviour: a peaceful outcome (grooming) or a conflict outcome (injury). We chose these two outcomes as they are mutually exclusive and represent plausible (and very different) outcomes from an approach. Based on previously published observational data documenting which social behaviours are likely to follow specific facial expressions in this species, we made the following predictions. We predicted that subjects would choose a grooming outcome when they see a neutral approach ending with a bared-teeth facial expression (Duboscq et al. 2013; Thierry et al. 1989). Contrastingly, we predicted that subjects would choose a conflict outcome when they see a neutral approach ending with a scream face or an open-mouth threat (Micheletta 2012; Thierry et al. 2000). Finally, we predicted that subjects would choose the two outcomes equally when the approach ended with a neutral face (control condition).

## Methods

### Subjects and housing

Subjects were three adult crested macaques (*M*. *nigra*). A single male (Bai, aged 9 years old) and two females (Sat, aged seven and Dru aged 12) housed within a social group of five individuals at the Macaque Study Centre, Marwell Zoo, Winchester, UK. All individuals had prior experience with touchscreens, with the matching-to-sample paradigm and had previous exposure to facial expression stimuli (Micheletta et al. 2015). All experiments were conducted in a specially built testing room (2 × 4 × 5 m), which was an extension to their normal enclosure and allowed unrestricted and voluntary access to the touchscreen area. The rest of the enclosure consisted of an indoor enclosure (5 × 5 × 4 m) an outdoor enclosure (10 × 5 × 4 m) and an island (15 × 15 m), all equipped with various enrichment devices including ropes, platforms, climbing structures, and puzzle boxes. Macaques were fed daily with assorted fruits and vegetables, nuts, seeds, and commercial monkey pellets. Water was available ad libitum. Tasks presented in this study were entirely voluntary, and macaques were free to participate at their own will. Animals were never deprived from food or water, and keeper schedules remained unchanged throughout testing. All experiments were performed in accordance with relevant guidelines and regulations. The procedures have been scrutinized and approved by the University of Portsmouth regulated Department of Psychology Ethics Committee.

### Apparatus and programming

All experimental programs were presented on a computerized touch screen (Elo 1939L 19-in. Open-Frame Touchmonitor, Elo Touch Solutions Limited, Swindon, UK). The touchscreen was linked to a laptop that ran the experiments (programmed using Visual Basic with Microsoft Visual Studio 2010). The programs recorded the identity of the individual participating in the task, the randomized order in which the stimuli were presented, the latency to complete a trial (from initiation of the trial, to their response), which response was chosen and if the response was correct or not. If participants left half way through an experimental session, progress was saved and resumed when the individual returned.

### Procedure

All tasks were conducted using an adapted version of the matching-to-sample (MTS) format (Parr and Heintz 2009; Micheletta et al. 2015). The macaques were required to attend to a touchscreen by touching a large cross, which was followed by a short video (5 s or less). At the end of the video, a sample image (the final frame from the video) appeared in a random central location (top, bottom, right, and left). In training trials, touching the sample image presented the animal with two choices: a correct ‘match’ choice or an incorrect ‘foil’ choice. Correct choices were reinforced by food rewards (cereal) and immediate transition onto the next trial (2 s), whereas incorrect choices initiated a delay onto the next trail (8 s) with no food reward. In test trials, touching the sample image presented the animal with two choices, both of which were reinforced by food rewards and led to immediate transition onto the next trial. Thus, during tests, spontaneous choices were reinforced. Subjects were tested twice per week opportunistically, and were presented a maximum of 48 trials per day.

### Stimuli

All stimuli presented were of unfamiliar individuals and were produced from images and videos taken from the Tangkoko Nature Reserve, North Sulawesi, Indonesia (Macaca Nigra Project field site: http://www.macaca-nigra.org), using digital SLR camera (Canon 50D mounted with a with a Canon EF 70–200 mm f/4 L USM) and a HD video camera (Panasonic HSC-SD700). Images were cropped into a square to reduce background information and increase the salience of the animal. Behavioural sequences were taken from longer videos to produce stimuli of 5 s or less. All photographic stimuli, including superimposing facial expressions onto the final video frame, were prepared through Adobe Photoshop CS5. All video stimuli were prepared through Adobe Premiere Pro CS5.

In the experimental portion of this study, we tested responses to three facial expressions: (1) bared-teeth: the upper and lower lips are both vertically retracted to present the teeth, which is often accompanied by the flattening of the ears and the raising of the scalp. This is an affiliative display, often used to initiate positive interactions (Thierry et al. 2000). (2) Open-mouth threat: mouth is open slightly, with corners drawn back—usually not accompanied by large teeth exposure, associated with aggression, and when displayed with non-vocal components (e.g. lunging) acts as a mild threat (Thierry et al. 2000). (3) Scream: mouth is open wide, with gums and teeth exposed. This expression is usually accompanied by vocalizations and staring. Screams are used during aggressive interactions and sometimes associated with counter-attacks (Thierry et al. 2000).

### Training phase

Animals were required to pass a training phase before progressing to the experimental phase. The purpose of this training phase was to ensure the individuals were conforming to our experimental rule (matching video sequences with the most likely possible outcome) and to ensure the animals were not following any other possible rules in the task. During the training phase, the animals were presented with various video sequences of behaviour (sexual presentation, aggressive chase, grooming presentation, and foraging; Supplementary Figure 1). Macaques were required to match the end frame of the video with likely behavioural outcomes to the videos: (1) sexual presentation was matched with mating, the foil being a picture of two individuals not interacting. (2) Grooming presentation was matched with grooming, the foil being a picture of two individuals mating. (3) An aggressive chase was matched with aggression (grab or injury), the foil being affiliation (ventral embrace). Foraging was matched with feeding, the foil being not feeding (neutral). Subjects were considered to have passed this phase when their performances exceeded chance in a single session (binomial z score >64.14 % or 31/48 correct responses) and when performance could be generalized to a novel stimuli set. For more information, please see ESM.

### Experimental phase

**Fig. 1:**
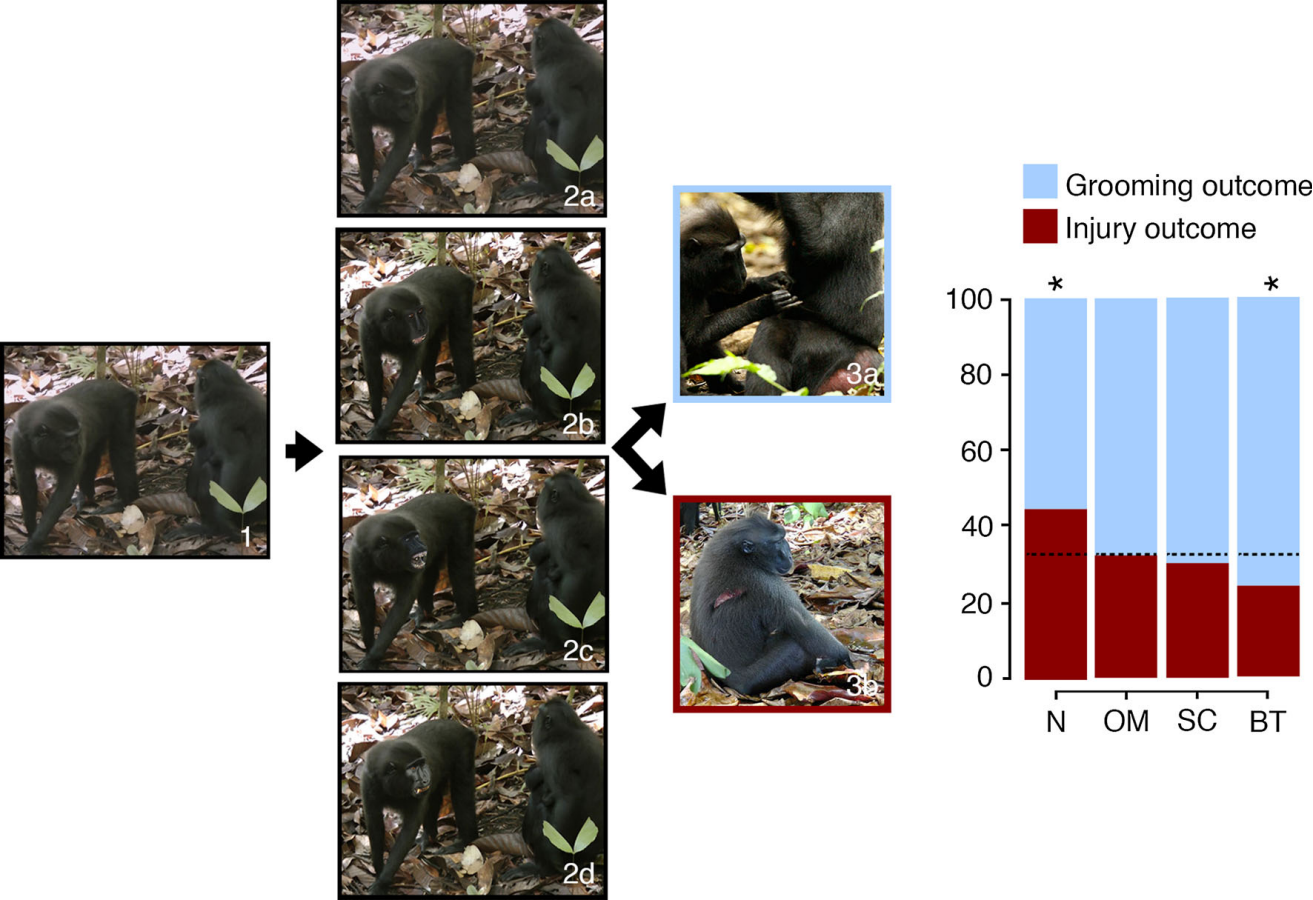
Graphical representation of the experimental procedure. Subject was presented with a video sequence of one unknown individual approaching another (*1*). The final frame of the video remained on the touchscreen, and the image was manipulated to either display: (*2a*) a neutral face (N), (*2b*) an open-mouth threat (OM), (*2c*) a scream face (SC), or (*2d*) a bared-teeth face (BT). Once the subject touched this image, the individual was then presented with a choice of social outcomes, (*3a*) grooming, or (*3b*) injury. The percentage of choice for each facial expression is given on the right panel. The *dotted line* represents the expected values derived from the Chi-squared analysis. Photographs taken by Jérôme Micheletta

One subject *Sat* progressed beyond the training (for more information about training and stimuli used, see ESM). In the experimental phase, *Sat* was presented with videos of one individual approaching another (see Fig. 1). The final frame was either kept neutral (as it was in the original video), or a facial expression was superimposed onto the macaque being approached (bared-teeth, open-mouth threat, or scream). The animal was then required to select one of two options: conflict-related injury or grooming. This experiment consisted of four unique approach videos, each with four unique facial expressions, matched against two unique matches and two unique foils (Supplementary Figure 2). This provided us with 64 unique trials. There were no pass criteria to this experiment, and instead, data were collected until six full sessions were achieved (384 trials).

## Results

We analysed *Sat*’s performance on the experimental trials (her choice of peaceful or conflict outcome) using Pearson’s Chi-square (facial expression × chosen social outcome) allowing within-subject comparison across trials. *Sat*’s choice of social outcome varied depending on the facial expression shown by the recipient of the approach in the social interaction sequence (*χ*^2^(3) = 9.97, *p* = 0.019). Post hoc pairwise comparison using Bonferroni-corrected *z* tests (Table 1) showed that *Sat* chose more peaceful outcomes when the recipient displayed a bared-teeth expression, suggesting *Sat* associated this expression with reduced likelihood of conflict. In contrast, *Sat* chose more injury outcomes than expected after neutral expressions. Thus, the absence of expression by the animals in the social interaction was associated more with a negative, conflict outcome. Adjusted residuals (Table 1) showed that *Sat* chose more injury outcomes after neutral expressions than any other expression, suggesting that to her all facial expressions (regardless of type) indicated that conflict was likely to be avoided.

**Table 1:** Data set subjected to analysis

Facial expression	Chosen social outcome		
	Grooming	Injury	Total
Bared-teeth	73 (2.1)^a^	23 (−2.1)^b^	96
Open-mouth threat	65 (0.1)^a^	31 (−0.1)^a^	96
Scream	67 (0.6)^a^	29 (−0.6)^a^	96
Neutral face	53 (−2.9)^a^	43 (2.9)^b^	96

Cross-tabulation subjected to Pearson’s Chi-square test (*χ* ^2^ (3) = 9.97, *p* = 0.019). Numbers represent frequency of choice by *Sat*. Numbers in parentheses represent adjusted residuals (residuals signify to what extent the value deviates from expected, with a residual of ±1.96 indicating significance). Letters represent the results of the post hoc pairwise comparison (Bonferroni-corrected *z* tests) where a difference in letters across chosen social outcomes indicates a significant difference

## Discussion

The findings show that macaques can use the information provided by conspecifics’ facial expressions to predict how social interactions might progress. Our subject chose more peaceful outcomes when the individual approaching displayed a bared-teeth expression. In contrast, she chose more injury outcomes when the individual displayed a neutral expression. However, and contrasting with our prediction, she did not choose more injury outcomes when the individual displayed an open-mouth threat or a scream face. Likewise, the absence of expression altogether was associated with greater potential for conflict than any of the facial expressions. Thus, *all* facial expressions indicated reduced potential for conflict, hinting at a social cohesion function of facial expressions in general. Our findings suggest that observers can use the facial expressions of others as pointers to potential actions, as signals of ‘action readiness’ (Frijda 2007) at least in some situations. Therefore, facial expressions might function as reliable predictive cues to the actions of others, reducing the uncertainty of the receiver. This ability could have enormous adaptive value when navigating a complex social environment with potentially limitless social outcomes.

We predicted that scream and open-mouth threat faces would be associated with injury outcomes (Thierry et al. 2000). However, the pattern of results did not meet this prediction, and these facial expressions were matched to the grooming outcome more often than the conflict outcome. Yet this finding might fit better with published data on the function of signalling in conflicts. A number of classic studies show that signalling can function to solve disputes without resorting to physical aggression (Clutton-Brock and Albon 1979; Davies and Halliday 1978; Preuschoft 1992). Indeed, in this species, only 20 % of aggressive acts result in physical retaliation (Duboscq et al. 2013), supporting the idea that facial expressions are used in aggressive contexts in order to *avoid* aggression.

A simple contextual association between the facial expression and the social outcome image cannot explain the findings, as macaques do not commonly produce any of the expressions used in the experiment, when grooming or when injured (Thierry et al. 2000). Instead, the subject must have understood the temporal association between the preceding behaviours and the social outcome and therefore *predicted* social outcomes.

As with all studies with only one experimental subject, we should be careful not to draw strong conclusions. However, this study constitutes a proof of concept showing that, at least in some cases, facial expressions can reveal tangible information about future events, which is an alternative to the mainstream interpretation that facial expressions reveal internal state alone. Humans are experts at navigating complex social worlds, in the past, present, and future. Whether nonhuman animals are capable of thinking beyond the present is intensely contested (Osvath and Martin-Ordas 2014). If, as shown by our results, the facial signals of others offer observers cues to the senders’ future actions, social communication is not constrained to the present.

## Electronic supplementary material

Below is the link to the electronic supplementary material.

Supplementary material 1 (PDF 56510 kb)
