# Supplementary material for: Macaques can predict social outcomes from facial expressions
Source: Anim Cogn. 2016 May 7;19:1031–6. doi: 10.1007/s10071-016-0992-3 (PMC4967087; doi:10.1007/s10071-016-0992-3)

Electronic Supplementary Information

Macaques can predict social outcomes from facial expressions

Bridget M. Waller[1], Jamie Whitehouse[1] and Jerome Micheletta[1]

[1] Centre for Comparative and Evolutionary Psychology, Department of Psychology, University of Portsmouth

Training

Animals were required to pass a training phase before progressing to the experimental phase. The purpose of this training phase was to ensure the individuals were conforming to our experimental rule (matching video sequences with the most-likely possible outcome), and to ensure the animals were not following any other possible rules in the task. Stimuli sets often consisted of videos that were matched with images with conflicting background information/number of individuals. Therefore, progression passed this phase would suggest the animals were following our predefined rule and not using these other potential cues to make their decisions.

During the training phase, the animals were presented with various video sequences of behaviour (sexual presentation, aggressive chase, grooming presentation and foraging; Supplementary Figure 1). Macaques were required to match the end frame of the video with likely behavioural outcomes to the videos: 1) Sexual presentation was matched with mating, the foil being a picture of two individuals not interacting. 2) Grooming presentation was matched with grooming, the foil being a picture of two individuals mating. 3) An aggressive chase was matched with aggression (grab or injury), the foil being affiliation (ventral embrace). Foraging was matched with feeding, the foil being not feeding (neutral).

The training experiment consisted of 12 unique trials repeated 4 times within a session (48 trials in total). Subjects were considered to have passed this experiment when their performances exceeded chance in a single session (binomial z-score > 64.14 % or 31/48 correct responses). *Sat* performed above chance on the 15th session, *Dru* did not perform above chance in 18 sessions and *Bai* did not perform above chance in 4 sessions. The training experiment was repeated with a new stimuli set to generalise the results of *Sat,* and to provide new (perhaps better) stimuli to increase motivations of the others. In the second set, *Sat* performed above chance on the 9th session, *Dru* did not perform above chance in 10 sessions and *Bai* did not perform above chance in 5 sessions. *Sat* progressed to the experimental phase, both *Dru* and *Bai* stopped engaging with the tasks.

Supplementary Figure 1

Examples of stimuli used in the training phrases. After presentation of a video of behaviour (sample), subjects were offered two choices, a likely outcome (match) or an unlikely outcome (foil). Four different behavioural sequences were presented to the subjects; 1) Sexual presentation was matched with mating, the foil being a picture of two individuals not interacting. 2) Grooming presentation was matched with grooming, the foil being a picture of two individuals mating. 3) An aggressive chase was matched with aggression (grab or injury), the foil being affiliation (ventral embrace). Foraging was matched with feeding, the foil being not feeding (neutral). Throughout the training, subjects were presented with 6 unique sets of stimuli for each behavioural sequence (72 unique stimuli, total).

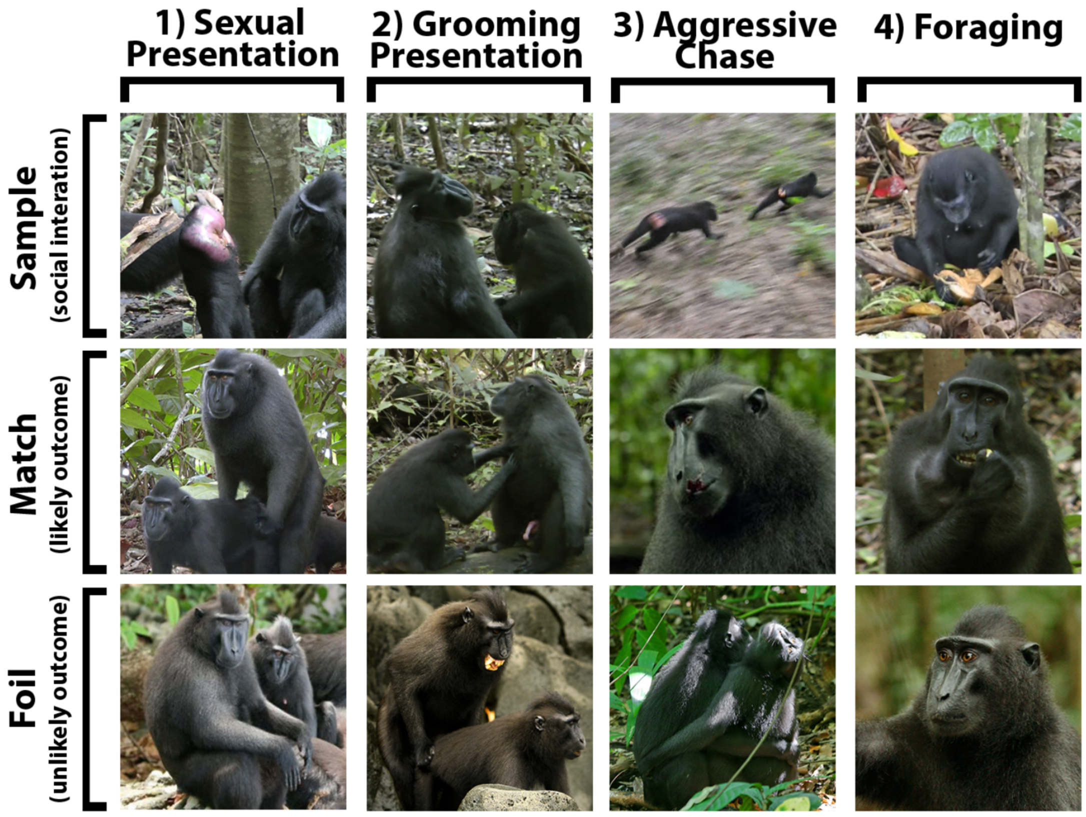

Supplementary Figure 2

Pictorial stimuli used in the experimental trials. Four video sequences, each having four possible expression stimuli (Neutral face, bared-teeth, open-mouth threat or scream), and the four possible social outcomes (conflict related injury x2, and grooming x 2).

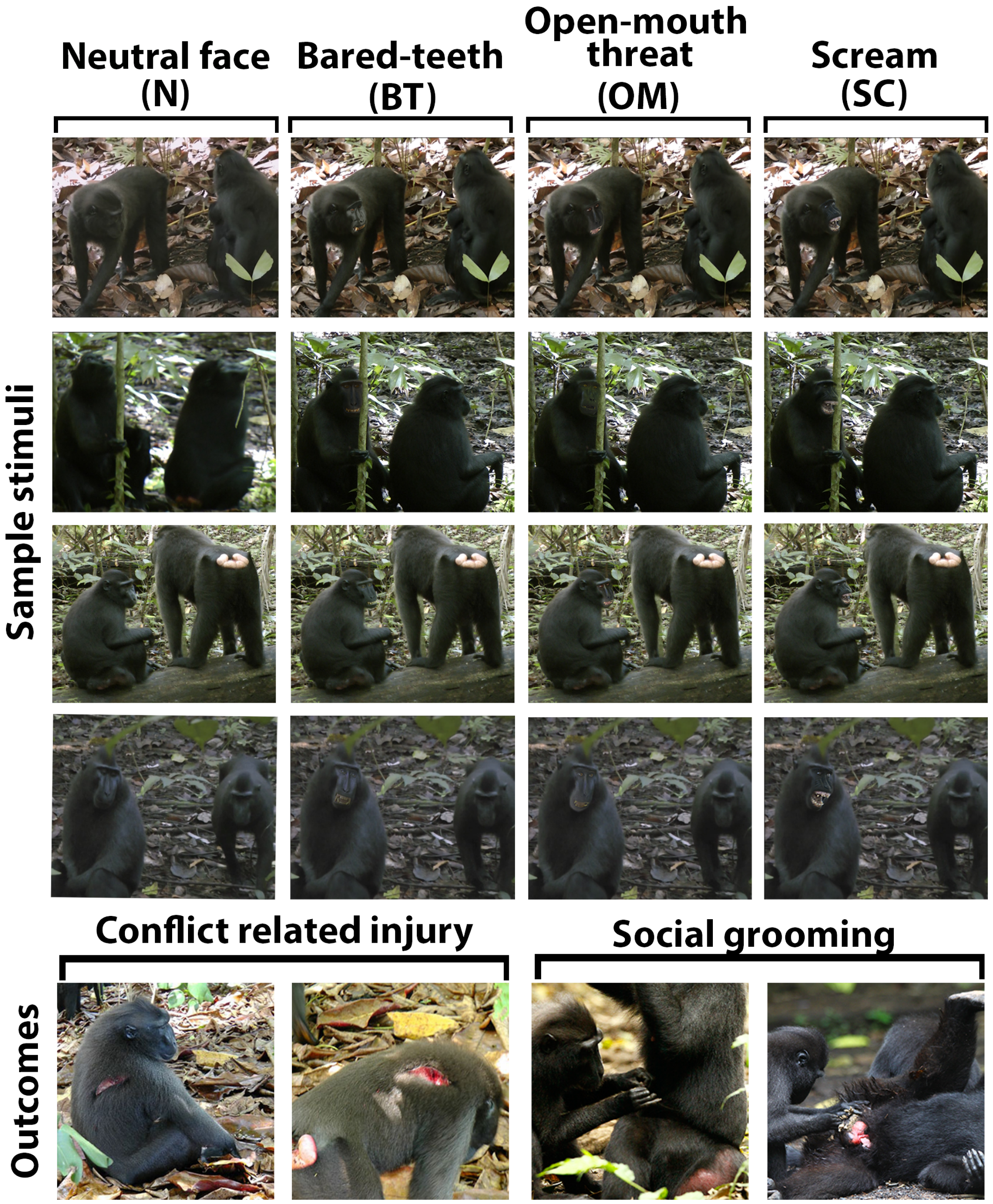

Supplement: Supplementary file 1 — Supplementary material 1 (PDF 56510 kb) [file 10071_2016_992_MOESM1_ESM.pdf]
